# The Video Head Impulse Test (vHIT) of Semicircular Canal Function – Age-Dependent Normative Values of VOR Gain in Healthy Subjects

**DOI:** 10.3389/fneur.2015.00154

**Published:** 2015-07-08

**Authors:** Leigh A. McGarvie, Hamish G. MacDougall, G. Michael Halmagyi, Ann M. Burgess, Konrad P. Weber, Ian S. Curthoys

**Affiliations:** ^1^Neurology Department, Institute of Clinical Neurosciences, Royal Prince Alfred Hospital, Camperdown, NSW, Australia; ^2^Vestibular Research Laboratory, School of Psychology, The University of Sydney, Sydney, NSW, Australia; ^3^Department of Neurology, University Hospital Zurich, Zurich, Switzerland; ^4^Department of Ophthalmology, University Hospital Zurich, Zurich, Switzerland

**Keywords:** vestibular, vestibulo-ocular reflex, VOR, vHIT, HIT, head impulse test, balance, bilateral vestibular loss

## Abstract

**Background/hypothesis:**

The video Head Impulse Test (vHIT) is now widely used to test the function of each of the six semicircular canals individually by measuring the eye rotation response to an abrupt head rotation in the plane of the canal. The main measure of canal adequacy is the ratio of the eye movement response to the head movement stimulus, i.e., the gain of the vestibulo-ocular reflex (VOR). However, there is a need for normative data about how VOR gain is affected by age and also by head velocity, to allow the response of any particular patient to be compared to the responses of healthy subjects in their age range. In this study, we determined for all six semicircular canals, normative values of VOR gain, for each canal across a range of head velocities, for healthy subjects in each decade of life.

**Study design:**

The VOR gain was measured for all canals across a range of head velocities for at least 10 healthy subjects in decade age bands: 10–19, 20–29, 30–39, 40–49, 50–59, 60–69, 70–79, 80–89.

**Methods:**

The compensatory eye movement response to a small, unpredictable, abrupt head rotation (head impulse) was measured by the ICS impulse prototype system. The same operator delivered every impulse to every subject.

**Results:**

Vestibulo-ocular reflex gain decreased at high head velocities, but was largely unaffected by age into the 80- to 89-year age group. There were some small but systematic differences between the two directions of head rotation, which appear to be largely due to the fact that in this study only the right eye was measured. The results are considered in relation to recent evidence about the effect of age on VOR performance.

**Conclusion:**

These normative values allow the results of any particular patient to be compared to the values of healthy people in their age range and so allow, for example, detection of whether a patient has a bilateral vestibular loss. VOR gain, as measured directly by the eye movement response to head rotation, seems largely unaffected by aging.

## Introduction

The development of objective measurements of the vestibulo-ocular reflex (VOR) in response to natural values of head angular acceleration – the video head impulse test (vHIT) (1) – has been valuable for identifying horizontal semicircular canal loss, either unilateral or bilateral. Recently, vHIT has been extended to testing vertical canal function, allowing fast simple and accurate assessment of the functional status of each of the six semicircular canals individually (2–4). vHIT has been validated by direct simultaneous comparison of vHIT with scleral search coils on the same eye of healthy subjects and patients with known vestibular loss, including those after unilateral vestibular schwannoma operations (1, 3). The usual measure of performance has been the gain of the VOR, and some values of normal VOR gain, averaged across healthy subjects, have been published (2, 3). However, these values have limitations: they are based on a relatively small number of subjects; they are single VOR gain values and so do not show VOR gain across a range of head velocities; and they do not show VOR gain at various age ranges. The present study sought to overcome these limitations.

The magnitude of the peak head velocity used in vHIT testing is important. Studies with scleral search coil recordings have shown that the value of VOR gain depends on the peak head velocity: for a given subject or patient, as the peak head velocity increases, VOR gain declines (5). However, patients with bilateral vestibular loss or patients with acute unilateral loss manifest their loss of semicircular canal function even at low head velocities, both by very low compensatory eye velocities and by the presence of corrective saccades accompanying such inadequate performance (5, 6). However, for other patients, the VOR gain may appear to be normal at low head velocities, and the loss becomes clear only as the peak head velocity is increased (5). For this reason, the value of VOR gain at just one head velocity is not an acceptable representation of vestibulo-ocular performance, so it is presently recommended that vHIT testing should include peak head velocities >150°/s (5, 7). Ideally, the values of VOR gain across a range of head velocities are needed, and the present study reports these.

In the present study, we used vHIT to measure VOR gain for each of the six semicircular canals, across a range of head velocities, in 91 healthy, community living subjects across a wide age range. This allowed us to specify the average VOR gain across many head velocities, at each decade of life, from 10–19 to 80–89, together with bands of ±2 SDs, which include 95% of the healthy population (8).

## Materials and Methods

### Subjects

All test subjects were healthy, independent, community-dwelling individuals with no history of any vestibular disorder. They were recruited from hospital staff and their associates as well as community groups for seniors. At least 10 subjects in each age decade from 10 to 90 without any prior known or reported balance problems were recruited and tested and the numbers and genders in each band are given in Table 1. They provided written informed consent. The study was conducted in accordance with the ethical standards of the Helsinki Declaration of 1975, as revised in 1983. The procedure was approved by the Human Ethics Committee of RPA Hospital Protocol number X11-0085.

**Table 1:** **Subject numbers and genders**.

Age range	Number of males	Number of females	Total number
10–19	5	5	10
20–29	3	7	10
30–39	3	8	11
40–49	4	8	12
50–59	9	3	12
60–69	5	6	11
70–79	7	8	15
80–89	0	10	10

*The number and gender of subjects tested in each decade age band*.

**Figure 1:**
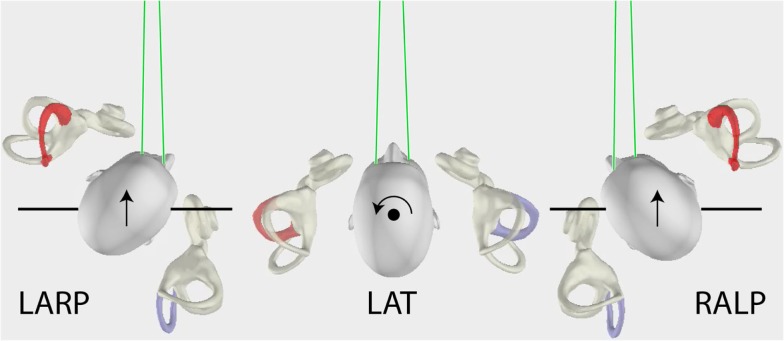
**The head movements for LARP (left anterior–right posterior) and RALP (right anterior–left posterior) and lateral semicircular canal stimulation (arrows), as viewed from above**. For testing the vertical canals, the person’s head is turned as shown and the movement of the head is a pitch rotation in the plane of the named canals as represented by the arrows. Note especially that the gaze position for testing the vertical canals is important (9) – it must be close to being along a line in the plane of the stimulated canal pair as shown by the green lines.

The measurement of horizontal VOR by vHIT has been described in detail previously (1, 7). Briefly, the subject was instructed to maintain the fixation on an earth-fixed target, which is usually straight ahead, while the operator delivered brief, passive head turns, which were unpredictable in size, direction, velocity, and timing. The application of vHIT to the measurement of vertical VOR (2, 3, 9) poses problems not encountered in testing horizontal canal function. The vertical canals are oriented in planes about 45° to the median plane of the head (10) and form two canal pairs – left anterior–right posterior (LARP) and right anterior–left posterior (RALP) (see Figure 1) – and to test these canals, the head impulses must be delivered in the plane of the canal pair under test. However, in these tests, to have a valid measure of vertical VOR, gaze must be directed close to a plane parallel to the canal pair under test (9) as shown in Figure 1.

### Eye movement recording

Video head impulse test tests were all carried out with prototype ICS impulse video goggles (GN Otometrics, Taastrup, Denmark), with a camera speed of 250 frames/s, recording motion of the right eye. Subjects were tested in a well lit room (to ensure a small pupil) with an eye-level target at a minimum distance of 1 m in front of them. Each subject was seated in a height-adjustable, rotatable office chair, so that their head was located at the ideal height for the operator to deliver horizontal or vertical impulses. If worn, the subject removed their spectacles. For results to be valid, vHIT goggles slippage must be minimized, and so the vHIT goggles were tightened on the head until movement of the goggles at the bridge of the nose was an absolute minimum, as tested by a gentle lateral tug on the goggles by the operator. With some subjects who did not have a prominent nose bridge, a firm foam insert was used to fill the gap between the goggles and the bridge of the nose to ensure goggle movement was a minimum. The room was illuminated and the fixation target height was selected to ensure that there were no reflections onto the pupil image at any point in the range of the head movement.

Calibration of the eye position signal was carried out with the subject successively fixating on two projected laser dots separated by a known horizontal angle. We verified that the X and Y axes of the camera chip had the same pixel spacing (the aspect ratio of the camera was 1.0), so the horizontal eye position calibration automatically also calibrated the vertical eye position. This has been verified in our previous paper (3). The calibration was also checked in each canal plane by an *“in vivo”* calibration – a slow sinusoidal motion of the head (about 0.3 Hz) in the plane of the canal while the subject fixated on the central fixation target. In this way, at low predictable head velocities, visual fixation ensures that the eye and head velocity traces should overlay. Eye velocity was obtained by differentiation of the eye position signal as we have explained in detail in our previous papers (1, 3). For each of the three canal planes, the head velocity signal used in the processing was the component of three-dimensional head velocity as measured by the sensor set in the plane of the test. For example, in the LARP plane, the head velocity signal is that measured by the sensor in the LARP orientation.

For each of the canal planes, the operator aimed to deliver a range of velocities in random order and direction so as to achieve at least 10 artifact-free impulses in each of the following ranges: horizontal: 10 <120°/s, 10 in the range 120–180°/s, and 10 over 180°/s in each direction. For vertical impulses, the ranges were: 10 <110°/s; 10 between 110° and 140°/s; 10 >140°/s. These arbitrary velocity ranges were selected to simplify statistical analysis. In practice, this meant that 50–60 impulses were delivered in each canal direction to ensure that the desired number of artifact-free responses was achieved.

### Horizontal vHIT

The horizontal vHIT stimulus consisted of the operator delivering a small, passive, abrupt horizontal head rotation, with an unpredictable direction and magnitude and with minimal “bounce-back” at the end of the head impulse: each impulse was a short sharp “turn and stop”. All tests were performed by the same right-handed operator (LAM). Horizontal tests were performed with both hands on the top of the head, well away from the goggles strap and forehead skin.

### Verticals vHIT

To test the LARP pair of canals, the subject was rotated *en bloc* to a position where the mid-sagittal plane of the body and head were pointed 30°–40° to the right of the fixation point. The subject was instructed to fixate the central fixation point eccentrically. To do that, the eye must look out the left corner of the orbit, and look along an earth-horizontal line close to being parallel to the plane of the LARP canal pair under test. With this eye position, the compensatory eye movement for stimulation of the vertical canals is an almost purely vertical eye movement (9, 11) (see Figure 1). Thus, a diagonal head pitch forward (toward the fixation target) activates the left anterior canal and causes an upward eye movement, and a head pitch back (away from the fixation target) activates the right posterior canal and causes a downward eye movement. In similar fashion, to test the RALP pair of canals, the seated patient was rotated *en bloc* so the head and body were pointed about 30°–40° to the left of the target, and the eyes were shifted to the right in the orbit (Figure 1). In this position, a head pitch forward activates the right anterior canal, and a head pitch back activates the left posterior canal. The whole test of the three canal pairs usually took only 10–15 min, and for the subject, this is not tiring since each impulse is so brief, and alertness is ensured by the random presentation of the stimuli.

#### Neck Stiffness

Part of the initial check in each plane was the slow sinusoidal head turn in order to ensure overlay of head and eye velocity traces. As well as a check of the system, this also allowed the operator to check the full range and ease of movement of the subject’s head. If there was any restriction or discomfort on the part of the subject, the range of movement was modified to avoid it. In order to minimize neck strain, the whole body was rotated into the LARP and RALP planes, rather than just rotating the head on trunk, to minimize patient discomfort. As the range of head rotation was small, the head impulses were usually well tolerated.

### Data processing

Prior to any gain calculations, each individual head and eye velocity record was checked to ensure that an acceptable head impulse movement was achieved, free of any artifact (12). In the head velocity component, this meant no head movement prior to the impulse, and as abrupt a stop as possible with a maximum “bounce-back” velocity of <25% of peak head velocity. For the eye velocity, it meant no eye movement prior to the onset of the head movement, and no obvious goggle movement or eyelid artifacts. Any impulse with these artifacts was eliminated prior to gain calculations. The remaining average number of impulses in each direction after removing the traces with artifacts ranged from a low of 32 impulses per subject (right anterior, 50–59 age group) to a maximum of 57 impulses per subject (right horizontal, 80–89 age group).

Vestibulo-ocular reflex gain was calculated as follows. The time of peak onset head acceleration was determined for each impulse (2), and head impulse onset was defined as occurring 60 ms before this time. Head impulse offset was defined as the moment when head velocity crossed zero velocity again (1, 3). Following the methods previously described (1, 3), the eye velocity time series were first desaccaded: saccades were identified by an eye acceleration criterion, and linear interpolation was used to replace the removed saccade. Then, the area under the desaccaded eye velocity curve from the start to the end of the head impulse was calculated and compared to the area under the head velocity curve during the same interval. VOR gain was defined as the ratio of these two areas. This is a position gain rather than the traditional slope gain (velocity) calculation, because our measurements and simulations (3) have shown that this method of calculating VOR gain with vHIT is more resistant to artifact – due, e.g., to slippage of the goggles – than the instantaneous VOR gain calculations using velocity. It is also a more functional measure of vestibulo-ocular performance, as it is the eye position error at the end of the head impulse (how far fixation is from the fixation target), which is the driver for the corrective saccade in the case of vestibular loss. VOR position gain and the corrective saccades are complementary (5).

**Figure 2:**
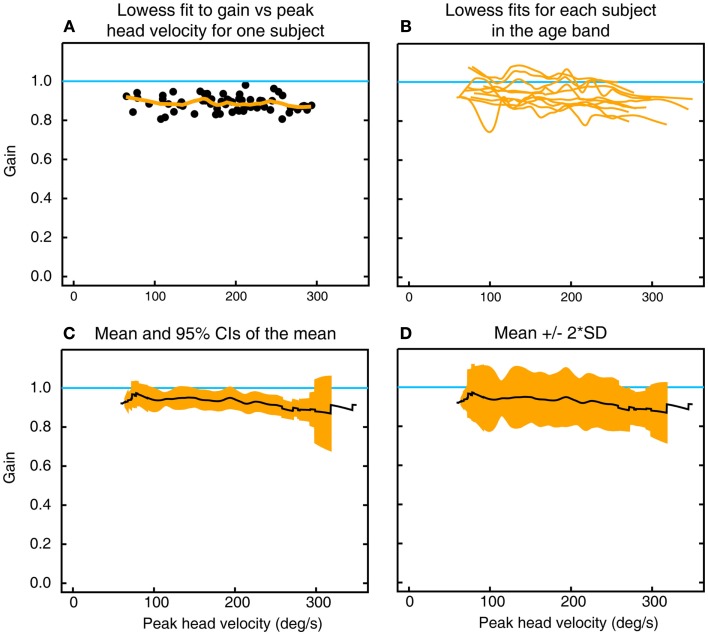
**To show the calculation of inter-subject means, confidence intervals, and SDs of gain as a function of peak head velocity**. **(A)** Gains for individual impulses (filled circles) as a function of peak head velocity for left horizontal canal stimulation in one subject in the decade 10–19 years, along with the lowess fit to these data (orange line). **(B)** Overlaid traces of lowess fits to gain as a function of peak head velocity for all 10 subjects in that age band. **(C)** Mean and two-tailed 95% confidence intervals of the mean for these data. **(D)** Mean ± 2 SD of the mean for the same data.

For each subject, the VOR gain of each impulse was plotted as a function of peak head velocity (Figure 2A). A line of best fit [using the lowess procedure (13) to perform a robust locally weighted regression] was fitted to those values using a smoothing fraction *f*, which depended on the range in head velocity covered, being chosen to correspond to an interval of 50°/s in peak head velocity. A cubic spline interpolation (using natural splines, where the second derivative was equal to zero at the endpoints) applied to the lowess-fitted data then provided a vector of VOR gain values at each 0.2°/s increment of peak head velocity (Figure 2A). The VOR gain vectors across all the subjects in that decade age group (Figure 2B) were averaged to form a vector of average VOR gain as a function of peak head velocity for that decade age group together with a band of ±95% confidence intervals of the mean (Figure 2C) and a band of the mean ± 2 SDs (Figure 2D) to show the band across velocities in which the results of 95% of the healthy population in that decade age group are expected to fall (8). This data processing procedure was repeated for each decade band for horizontal, anterior, and posterior canals. Plots of two-tailed 95% confidence intervals were important for showing whether the VOR data for an age band included the VOR gain of 1.0.

### Statistical analysis

Separate analyses of variance were carried out on the VOR gains for horizontal, anterior, and posterior canal VOR gains. In these analyses, the data for only the first 10 subjects in each decade’s age band were used. For the ANOVA, the VOR gains used were in each of the three bands defined above. For each subject, the VOR gains in the band were averaged to yield a single value in each band and the ANOVA was carried out on those averaged data. In this experiment, the data for leftward and rightward head impulses were analyzed for each of the canals separately in a mixed-design ANOVA with two repeated measures [impulse direction (leftwards, rightwards) and velocity (low, medium and high)] and one between-subject factor (age range). Shapiro–Wilk tests of normality showed that the assumption of normality of distribution of the raw data was accepted in all except two conditions, so no data transformation was carried out. The level of statistical significance was set at *p* < 0.05. Mauchly’s test of Sphericity (*W*) for velocity was significant for each of the canals, hence the Greenhouse and Geisser estimate was used to adjust the degrees of freedom for the *F*-ratio (14).

## Results

### Horizontal

**Figure 3:**
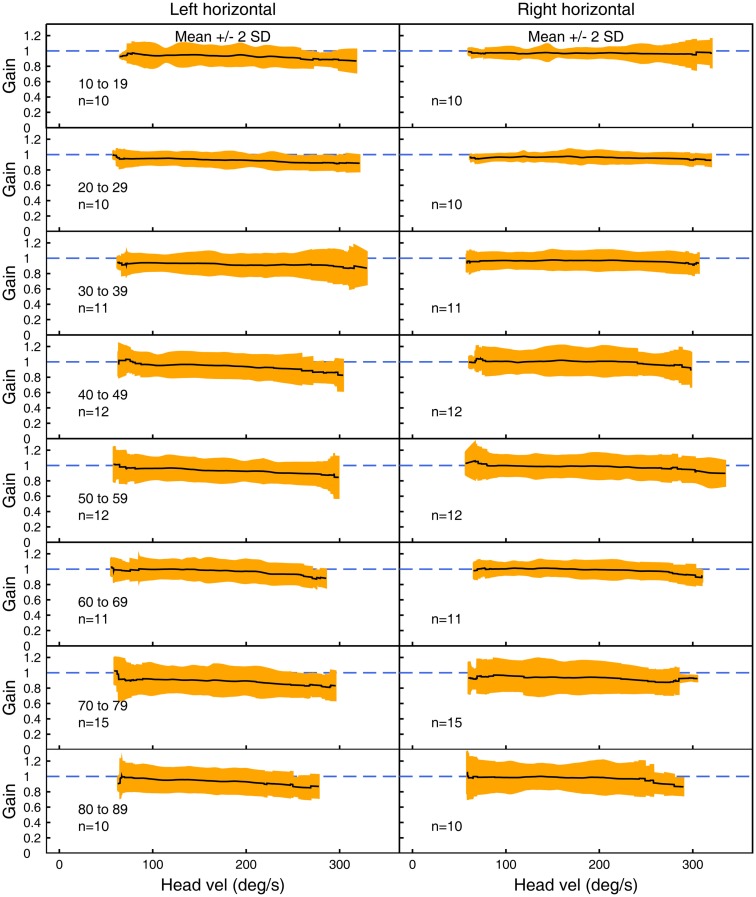
**The average VOR gain for left and right horizontal canal stimulation across velocities ± 2 SDs around the mean, for each decade age band**.

**Figure 4:**
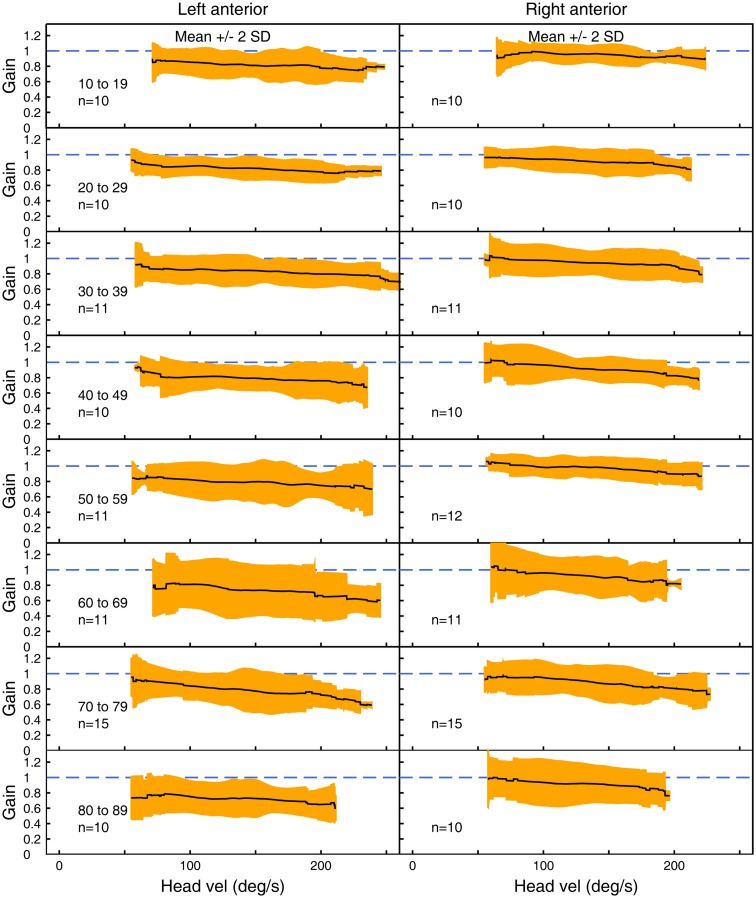
**The average VOR gain for left and right anterior canal stimulation across velocities ± 2 SDs around the mean, for each decade age band**.

**Figure 5:**
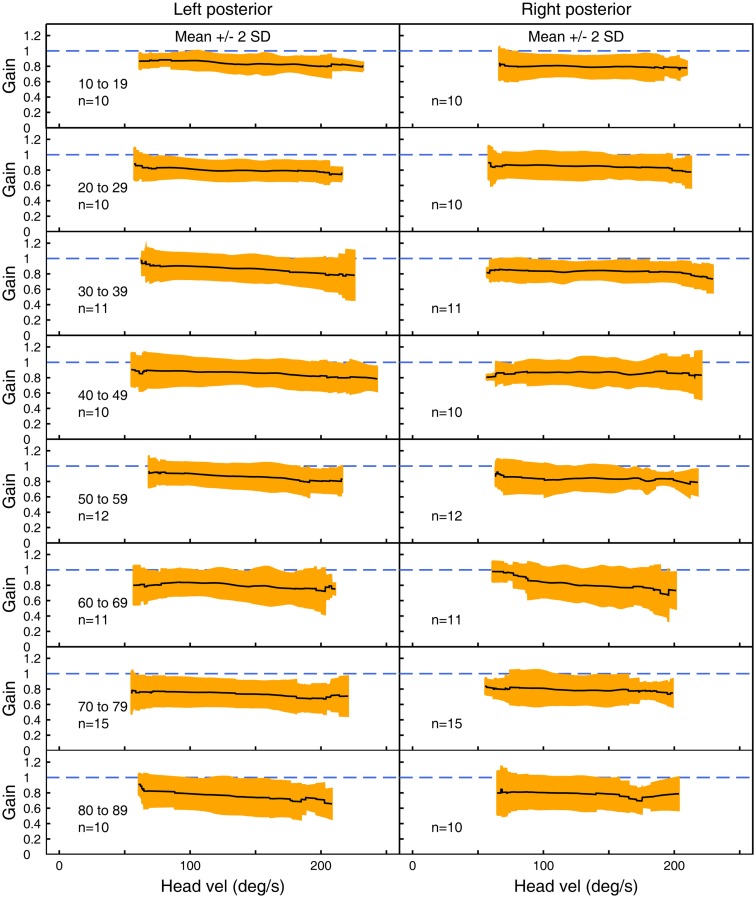
**The average VOR gain for left and right posterior canal stimulation across velocities ± 2 SDs around the mean, for each decade age band**.

Figures 3–5 show the means ± 2 SDs for VOR gain for horizontal, anterior, and posterior canals, respectively at each age band from 10–19 to 80–89, averaged across all velocities. Figure 3 shows that for the youngest subjects, horizontal VOR gain is tightly clustered around a gain of 1.0 at low velocities, with a small decrease in VOR gain as velocity increases. A similar pattern occurred for each decade band and for all canals (Figures 4 and 5). Variability of VOR gain was much greater in the vertical planes, with a more rapid drop-off in VOR gain as head velocity increased. For each decade age group, the results were similar, with the average VOR gain across velocities showing only a small decline with age.

**Figure 6:**
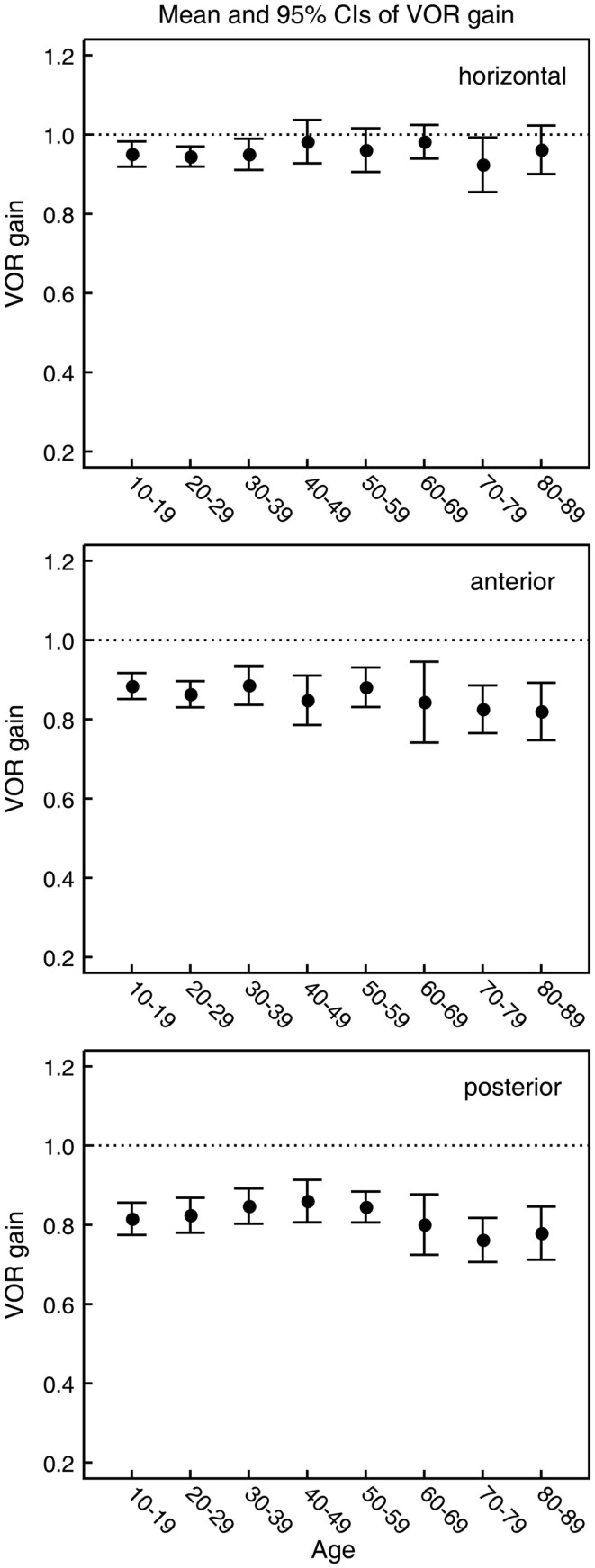
**Mean and 95% confidence intervals of VOR gain as a function of age for the horizontal, anterior, and posterior canals (top, middle, and bottom panels, respectively)**. Ten subjects were included for each decade of age. The within-subject mean VOR gain was calculated for each of the six semicircular canals over the whole range of peak head velocities. For horizontal, anterior, and posterior canals, within-subject means were then calculated from the mean VOR gain for that canal on the left and right side of the head. These values were then used to calculate the between-subject means over all 10 subjects. As shown by the ANOVA, the gains for horizontal canals and anterior do not vary significantly with age but the average gains for the posterior canals decrease.

In all three ANOVAs (Table S1 in Supplementary Material), the factor velocity was significant – at every age, there was a decrease in VOR gain as head velocity increased (Figure 6). For the horizontal and anterior canals, the factor Direction was significant. For all canals, we found little decrease in VOR gain with age, at least up to the 80s, such that the factor Age was not significant for horizontal and anterior canals and Age was only weakly significant (*p* < 0.02) for the posterior canal. To elaborate the cause of the significant factors, Figure 6 shows the data averaged across velocity in each decade band (mean ± 95% CIs). So for the horizontal canal, the two-tailed 95% confidence intervals for VOR gain include or are very close to 1.0 even for subjects in their 70s and 80s. For the anterior and posterior canals, the average VOR gain was significantly <1 at all ages (Figure 6).

There is a systematic difference between gains for left anterior and right anterior canals, with right anterior canals showing a small but systematically larger gain at all velocities tested. This appears to be an unintended consequence of the test protocol, in which only the right eye was measured. In conditions where there was a larger translation of the measured eye with respect to the target, the VOR gain was higher.

## Discussion

The bands provided here serve as templates against which the VOR results for any individual patient can be judged, by whether their results lie within the normal limits, which include 95% of the healthy population. Age was not a statistically significant factor in this large sample of healthy subjects into their 80s, except for a small decrease in the VOR gain for the posterior canal. This small decrease with age is in accord with another recent study using vHIT of horizontal VOR gain and age (15), and extends that finding to the vertical canals. These results are in contrast with studies using more indirect measures of VOR performance, such as VOR inferred from dynamic visual acuity (DVA) performance (16) where, at least in one sample, there was reported to be a significant decrease in VOR gains inferred from DVA, after about age 60. These issues are considered below.

### Subject selection

A major issue in studies, such as these, is subject selection. We stress that the subjects we selected were independent, healthy, community dwellers. We sought to have a reasonably large group in each age band that would serve as the healthy cohort for any patient. Other studies have recruited subjects from more restricted populations (16), so their results would be expected to differ from ours. There is one other important issue: in the present study, every head impulse to every subject was delivered by the same very well trained operator (LAM), who has been delivering head impulse tests for over 20 years. Developing baseline results such as this by combining data from different operators of varying skill level allows for the possibility of idiosyncratic differences between operators to confound the results.

### Age and vestibular function

There is a large body of evidence of loss of vestibular receptor cells and primary afferents with age. In healthy young adults, there is a very considerable number of receptors in each canal, but it is reported that this number declines with age (17–19), which may suggest that there would be declines of the VOR with age. Even the histologists who showed these receptor and neural declines were puzzled about why there were not more obvious functional deficits, as shown by the quote: “the present study has shown considerable degenerations inside vestibular sensory regions occurring as a result of increasing age. Still very many people, who without doubt have such reductions, behave in a rather accurate way from a functional point of view. This must mean that the vestibular sensory regions can act fairly well with a reduced population of both sensory cells and nerve fibers and with considerable waste products inside the epithelial cells” p. 418 (17). The oculomotor response measured here is controlled by the cerebellum, and much research on the VOR has shown how important the cerebellum is for “repairing” the VOR in the face of “challenges,” such as magnified vision or probably age (17, 20, 21). The relatively inconsequential effect of age we have shown may be due to such cerebellar repair.

Many studies have reported a decline in DVA with age [see Ref. (16) for a review]. However, DVA is an indirect measure of vestibular function – it is the threshold for recognizing a briefly flashed optotype during a head movement. Poor DVA has been interpreted as indicating poor semicircular canal function, based on the argument that an inadequate VOR will cause smear of the flashed letter across the moving retina and so degrade the recognition of that letter. While an inadequate VOR gain will indeed result in smearing of the image (depending on stimulus duration, head velocity, etc.) and so degrade visual acuity, there are other non-vestibular age-dependent factors, which can and probably do affect letter recognition in this paradigm – such as luminance (22, 23). Testing senior subjects without their optical correction would also impact their DVA performance. In light of our direct measures of VOR [and those of others (15)], we question the interpretation of the cause of that DVA decrement as being a unique indicator of decreased semicircular canal function. We do acknowledge that DVA is a very good indicator of decreased balance-related performance with age but, in light of the results above, we caution about interpreting the DVA decrease as being due solely to declining VOR function.

### Directional differences

Our results revealed some small but significant effects, which could be due to the fact that only the right eye was measured. The following considers possible causes for these small effects, but we stress that in clinical testing, the normative data shown in Figures 3–5 are the most relevant data for interpreting the results of any individual. The first result is that for the horizontal canal, the VOR gain for rightwards impulses is generally slightly (but statistically significantly) higher than the VOR gain for leftwards impulses. This result is consistent with the geometry of the test, and the fact that it was the right eye, which was measured. As the head rotates to the right, the right eye has to make a slightly larger rotation within the skull to remain fixated on a target 1 m from the subject, in comparison to the left eye. The effect is reversed for an impulse rotation to the left. This difference is a “demand” for a higher VOR gain for rightwards head turns. In previous studies where such “demands” are placed on the VOR by varying fixation distance, it has been shown that the VOR gain changes in accord with that demand (24). This effect has also been confirmed by measuring both eyes with dual search coils, which has shown up to 15% difference in the slope gain between the adducting and abducting eyes at high velocities during head impulses (25). We suggest that the small horizontal VOR gain difference for left and right that we measured is due to such different demands.

Similarly, for vertical responses (LARPs and RALPs), the data showed a systematic asymmetry – the mean left anterior canal VOR gain (LA) was systematically lower at all velocities than the mean right anterior canal VOR gain (RA). As for the horizontal responses, it seems that the underlying cause is due to measuring the right eye response, rather than any left–right difference in the semicircular canals. There are very different vertical translations of the measured right eye during LARP versus RALP stimulation, with the vertical translation being significantly less for left anterior impulses than for right anterior head impulses. The increased vertical translation of the right eye with respect to the target during right anterior impulses requires a greater corrective eye rotation to remain on target and, so would be expected to provide an increased gain of the VOR. Even though the angular acceleration remains the same, the “demanded” eye rotation increases. In this situation, the left anterior–right anterior VOR gain difference is the vertical analog of the horizontal gaze distance dependence (24). This matter will only be resolved when it will be possible to make exactly simultaneous video measures of both eyes during LARP and RALP stimulation. However, for clinicians using a video system measuring the right eye, the results here are the appropriate normative data for comparison.

### Need for high velocity in clinical testing

Impulses with low peak head velocity (below 100°/s) are, for most patients, not adequate tests of semicircular canal function since some patients with unilateral vestibular loss can generate eye velocities in the normal range at these low velocities [(5) Figure 3], so their VOR gain at low peak head velocities is in the normal range. This result is probably mediated by the disinhibition from the semicircular canals of the healthy ear (26–28). The exceptions to this rule are patients with bilateral vestibular loss and patients with acute vestibular deficits, where even very low head velocities clearly show the unilateral loss of canal function (29). We consider that the ideal vHIT test consists of a range of head velocities as we have used here. But it is important to stress that, in routine vestibular testing, there should be some high velocity impulses.

### Bilateral vestibular loss

The normative data presented here are particularly valuable for the identification and assessment of patients with moderate bilateral vestibular loss – for example, patients who have received (or are receiving) systemic gentamicin as a therapeutic procedure. One consequence of such therapy can be bilateral vestibular loss, and in some patients, even small doses can result in ototoxic loss (30, 31). If there is bilateral loss of semicircular canal function, there is no rotational vertigo, but when the patient attempts to walk again they have severe postural unsteadiness (6, 32, 33), which is long-lasting and probably permanent. Partial bilateral loss is difficult to identify by caloric testing because the range of normal caloric nystagmus values is so large that a small response may fall in the very wide normal range for caloric nystagmus. Such bilateral vestibular loss patients do not have an asymmetry of function so canal paresis scores are normal. With scleral search coils, we have shown how head impulse testing can identify bilateral vestibular loss in such patients (6) because head impulse testing shows the absolute value of the VOR gain. However, to interpret that absolute value, information about normative VOR gains for people in the same age range is needed, and it is provided here. vHIT closely matches search coil measures and so also clearly identifies bilateral vestibular loss (1, 3), and values systematically below the limits of the appropriate normative data for the age bands reported here are indicative of bilateral loss. Of particular importance is the fact that vHIT allows sequential testing, even on a daily (even hourly) basis, of patients receiving ototoxic antibiotic treatment to identify the extent to which semicircular canal function may be affected progressively by gentamicin ototoxicity.

### Limitations of the study

In order to present data from healthy, independent community-dwelling individuals, it was easier to find female participants than males, reflecting the natural gender distribution in elderly subjects – there are no males in the 80–89 cohort. Additionally, the distance to the fixation target was not tightly controlled. In order to minimize geometrical effects, a minimum distance of 1 m was used. However, as the testing locations varied, the maximum distance was not fixed and consequently the distance to the fixation target was likely to vary in the range 1–1.8 m. This variation is unlikely to affect the results, as a greater distance to the target has the effect of reducing geometrical effects.

## Conclusion

These normative values of VOR gain allow the results of any particular patient to be compared to the values of healthy people in the patient’s age range and so allow, for example, detection of whether a patient has a unilateral or bilateral vestibular loss. VOR gain, as measured directly by the eye movement response to head rotation, seems largely unaffected by aging.

## Author Contributions

LM, HM, GH, and IC designed the experiment, which was carried out by LM. LM, AB, and IC analyzed the data and prepared figures; all authors were involved in the interpretation of the data. IC wrote the paper, and all authors revised it.

## Conflict of Interest Statement

Leigh A. McGarvie, Hamish G. MacDougall, G. Michael Halmagyi, Ann M. Burgess, and Ian S. Curthoys are currently receiving a project grant (App1046826) from NHMRC of Australia. Ian S. Curthoys is currently receiving a project grant from the Garnett Passe and Rodney Williams Memorial Foundation (Grant RP228); this grant helps to pay the salary of Ann M. Burgess. Hamish G. MacDougall is currently receiving a grant from the Garnett Passe and Rodney Williams Memorial Foundation. Leigh A. McGarvie, Hamish G. MacDougall, G. Michael Halmagyi, and Ian S. Curthoys are unpaid consultants to and have received funding for travel from GN Otometrics. Konrad P. Weber has no conflict of interest to declare.

## Supplementary Material

The Supplementary Material for this article can be found online at http://journal.frontiersin.org/article/10.3389/fneur.2015.00154

## Abbreviations

95% CIs, two-tailed 95% confidence intervals for the mean; HIT, head impulse test; LARP, the plane of the left anterior–right posterior canals; RALP, the plane of the right anterior–left posterior canals; SD, standard deviation; vHIT, video head impulse test; VOR, vestibulo-ocular reflex.
