# Supplementary figures and images for: The Video Head Impulse Test (vHIT) of Semicircular Canal Function – Age-Dependent Normative Values of VOR Gain in Healthy Subjects
Source: Front Neurol. 2015 Jul 8;6:154. doi: 10.3389/fneur.2015.00154 (PMC4495346; doi:10.3389/fneur.2015.00154)

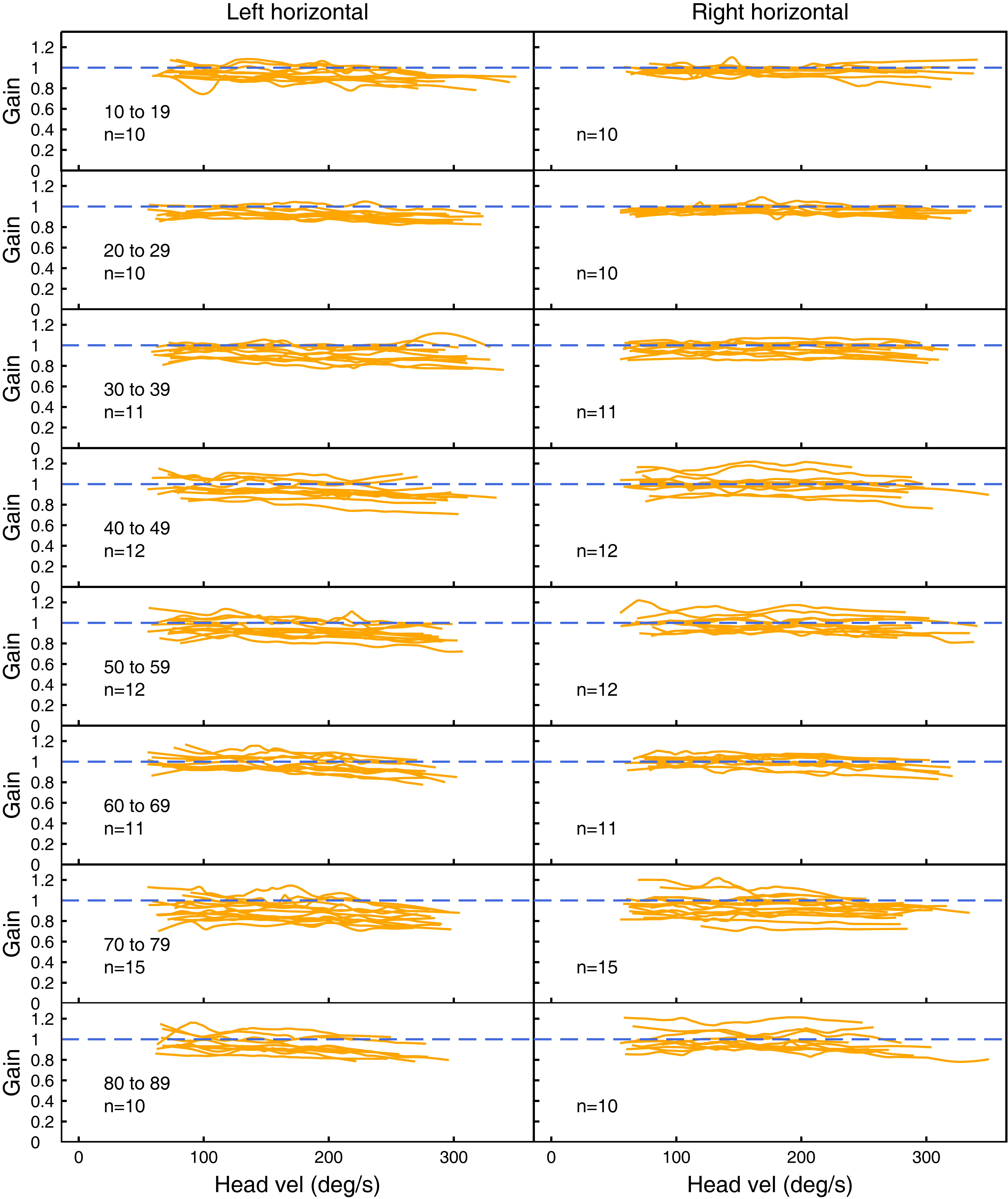

Supplement: Supplementary file 1 [file image_1.tif]

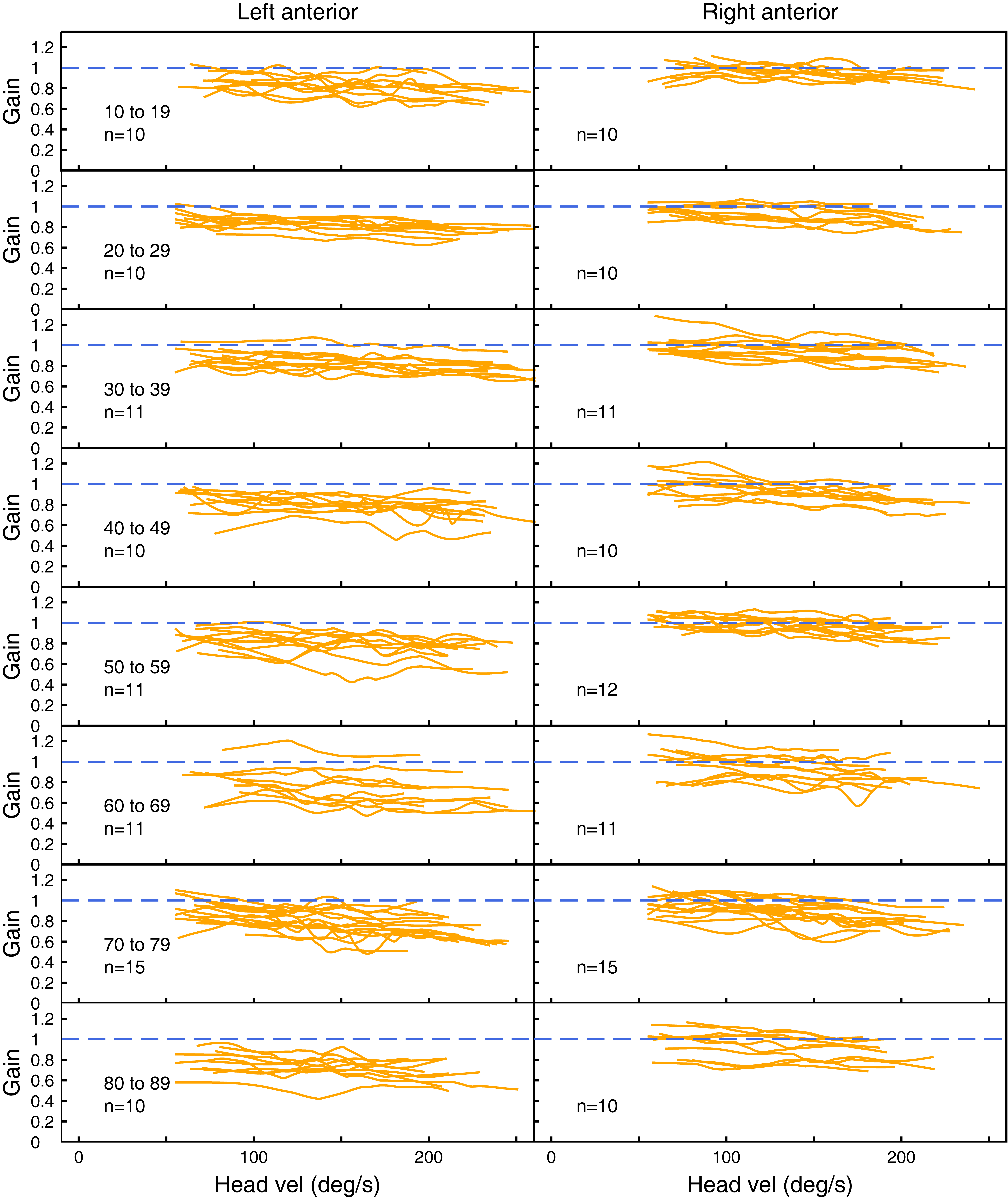

Supplement: Supplementary file 2 [file image_2.tif]

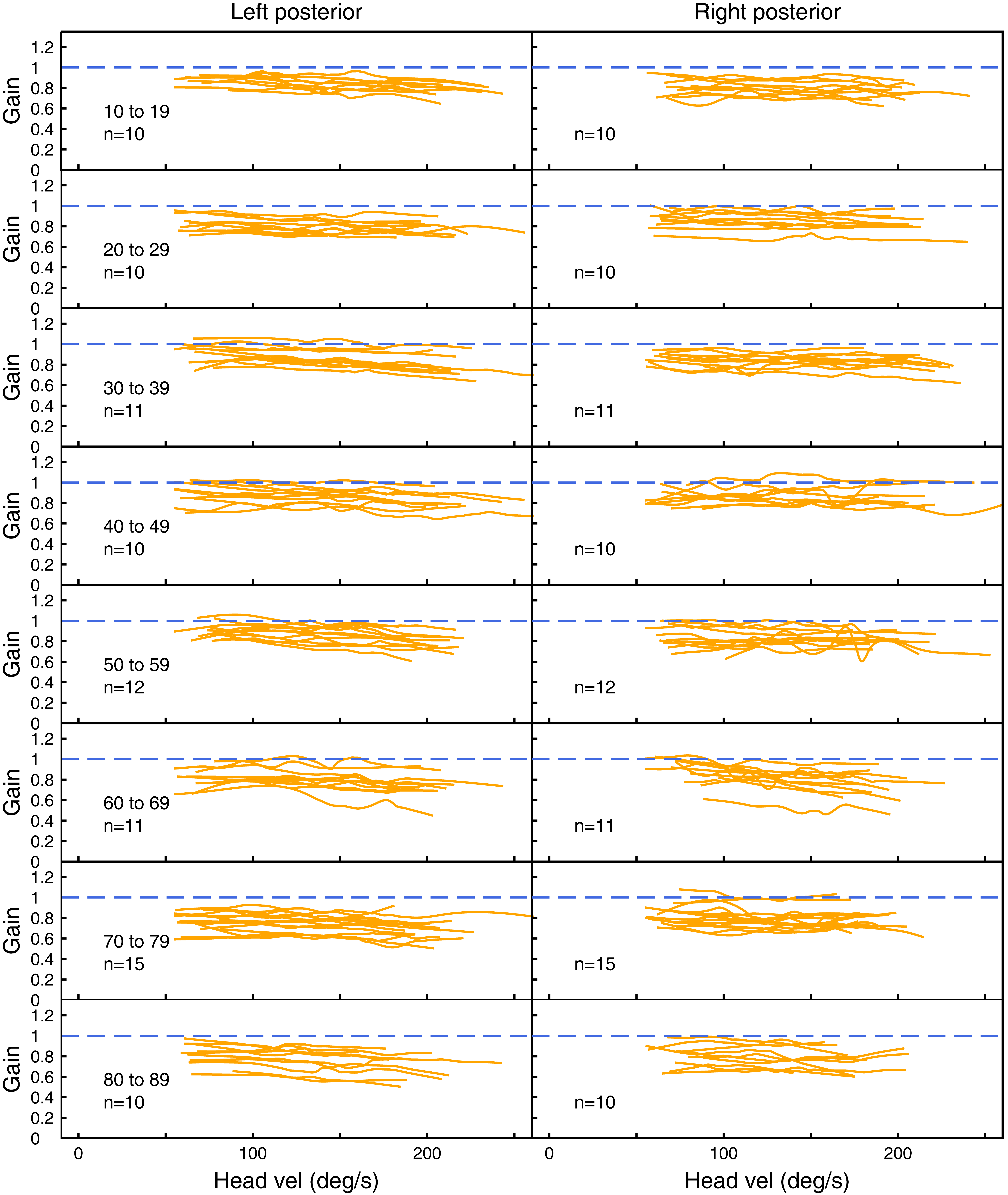

Supplement: Supplementary file 3 [file image_3.tif]

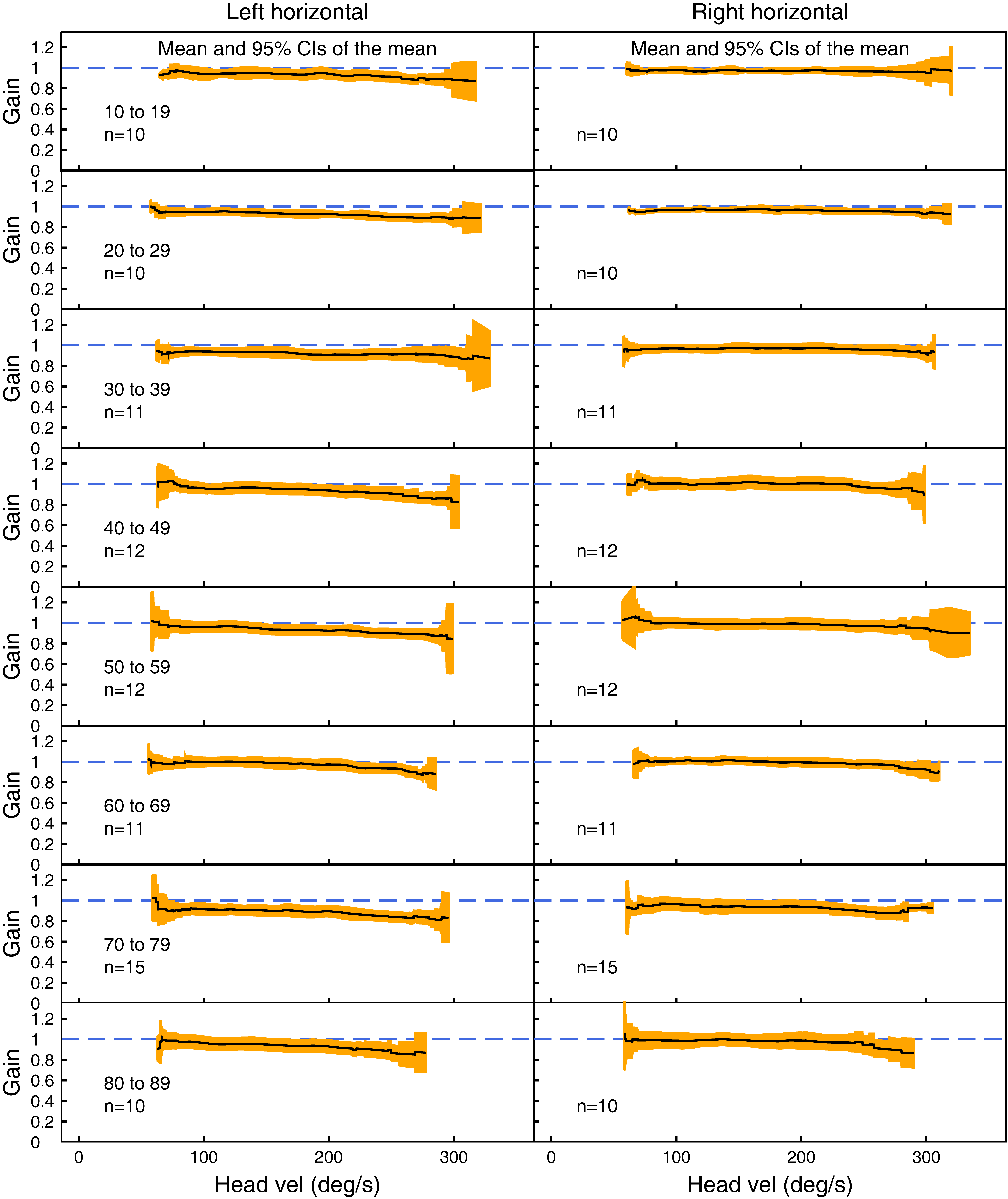

Supplement: Supplementary file 4 [file image_4.tif]

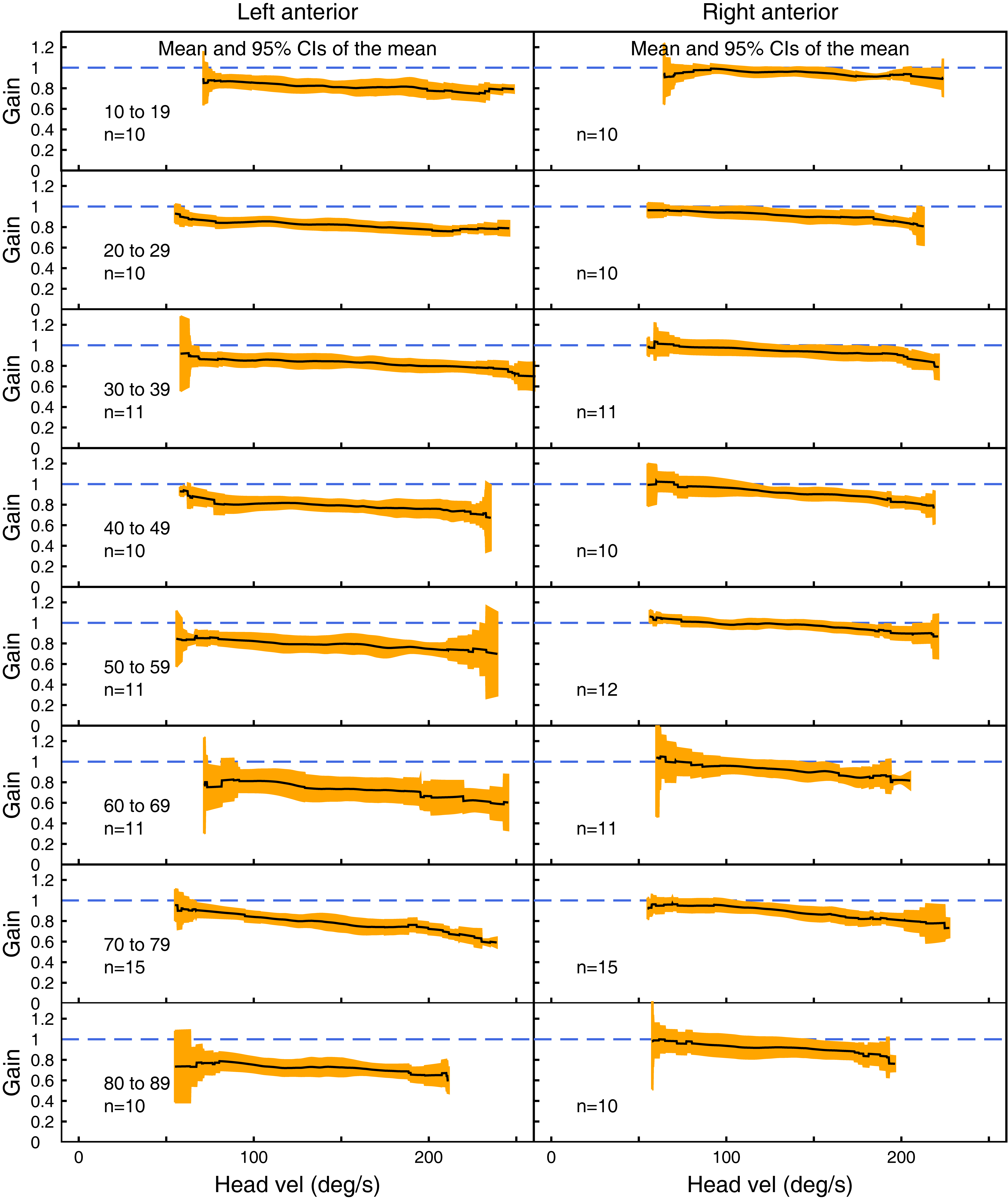

Supplement: Supplementary file 5 [file image_5.tif]

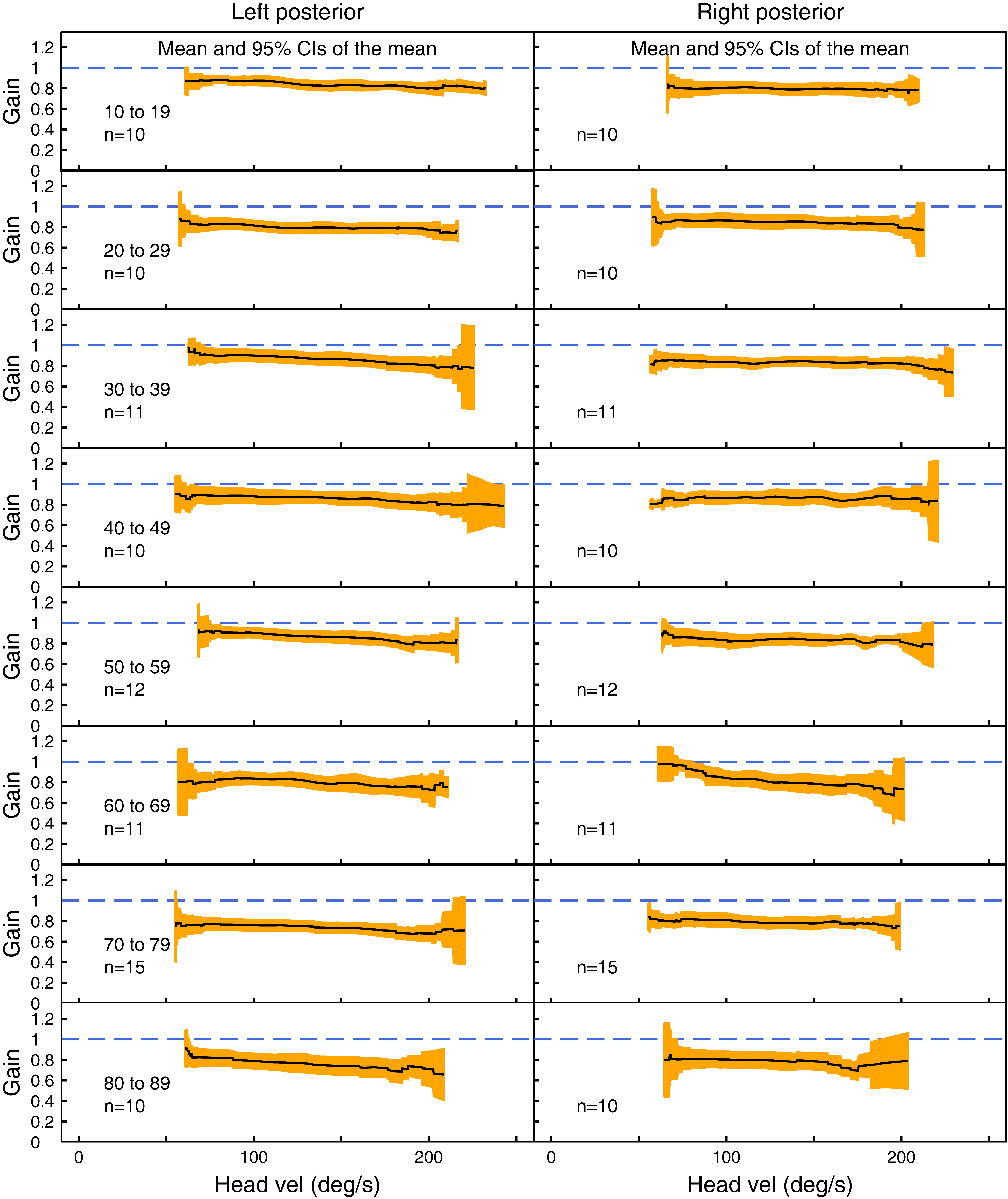

Supplement: Supplementary file 6 [file image_6.tif]
