# Supplementary material for: The Video Head Impulse Test (vHIT) of Semicircular Canal Function – Age-Dependent Normative Values of VOR Gain in Healthy Subjects
Source: Front Neurol. 2015 Jul 8;6:154. doi: 10.3389/fneur.2015.00154 (PMC4495346; doi:10.3389/fneur.2015.00154)
Supplement: Supplementary file 7 [file presentation_1.pdf]

Supplementary Material

The video head impulse test (vHIT) of semicircular canal function – age dependent normative values of VOR gain in healthy subjects

L. A. McGarvie[1], H. G. MacDougall[2], G. M. Halmagyi[1], A. M. Burgess[2], K. P. Weber[3], I. S. Curthoys[2]*

[1]Institute of Clinical Neurosciences, Neurology Department, Royal Prince Alfred Hospital, Camperdown, NSW, Australia
[2]Vestibular Research Laboratory, School of Psychology, University of Sydney, Sydney, NSW, Australia
[3]Departments of Neurology and Ophthalmology, University Hospital Zurich, Zurich, Switzerland

*** Correspondence:** I. S. Curthoys, Vestibular Research Laboratory, School of Psychology, Brennan MacCallum A18, NSW 2006, Australia.
ianc@psych.usyd.edu.au

1. Supplementary Table

Table S1. Analysis of Variance Results for Horizontal, Anterior, and Posterior canals respectively. Note: for the Within-Subject effects, those rows in which df (degrees of freedom) has an integer value, are those for which the Greenhouse-Geisser correction did not need to be applied.

Horizontal Canal

**WITHIN-SUBJECT EFFECTS Source**	**Sum of Squares**	**df**	**Mean Square**	**F**	**Sig**
**Impulse Direction**	.189	1	.189	66.253	.000
Error (direction)	.206	72	.003		
**Velocity**	.076	1.354	.056	67.317	.000
Error (velocity)	.081	97.490	.001		
**Impulse direction x Velocity**	.019	1.769	.011	39.007	.000
Error (direction x velocity)	.035	127.380	.000		
**BETWEEN-SUBJECT EFFECTS**					
**Age Range**	.175	7	.025	.950	.474
Error	1.898	72	.026		

Anterior Canal

WITHIN-SUBJECT EFFECTS Source	Sum of Squares	df	Mean Square	F	Sig
**Impulse Direction**	2.355	1	2.355	170.922	.000
Error (direction)	.992	72	.014		
**Velocity**	.404	1.559	.259	92.744	.000
Error (velocity)	.313	112.232	.003		
**Impulse direction x Velocity**	.000	2	.000	.187	.830
Error (direction x velocity)	.172	144	.001		
**BETWEEN-SUBJECT EFFECTS**					
**Age Range**	.290	7	.041	1.010	.432
Error	2.958	72	.041		

Posterior Canal

WITHIN-SUBJECT EFFECTS Source	Sum of Squares	df	Mean Square	F	Sig
**Impulse Direction**	.000	1	.000	.034	.855
Error (impulse direction)	1.015	72	.014		
**Velocity**	.122	1.472	.083	66.752	.000
Error (velocity)	.131	105.962	.001		
**Impulse direction x Velocity**	.020	1.496	.013	11.511	.000
Error (direction x velocity)	.125	107.697	.001		
**BETWEEN-SUBJECT EFFECTS**					
**Age Range**	.542	7	.077	2.429	.027
Error	2.296	72	.032		

2. Supplementary Figures

Figure S2. Overlaid traces of the average VOR gain for left and right horizontal canal stimulation as a function of peak head velocity. Each trace represents a lowess fit to the data for one subject.

Figure S2. Overlaid traces of the average VOR gain for left and right anterior canal stimulation as a function of peak head velocity. Each trace represents a lowess fit to the data for one subject.

Figure S3. Overlaid traces of the average VOR gain for left and right posterior canal stimulation as a function of peak head velocity. Each trace represents a lowess fit to the data for one subject.

Figure S4. Mean (black line) and 95% confidence intervals (orange bands) of VOR gain for left and right horizontal canal stimulation as a function of peak head velocity. These were calculated from the data shown in Figure S1.

Figure S5. Mean (black line) and 95% confidence intervals (orange bands) of VOR gain for left and right anterior canal stimulation as a function of peak head velocity. These were calculated from the data shown in Figure S2.

Figure S6. Mean (black line) and 95% confidence intervals (orange bands) of VOR gain for left and right posterior canal stimulation as a function of peak head velocity. These were calculated from the data shown in Figure S3.
